# Free circulating mircoRNAs support the diagnosis of invasive aspergillosis in patients with hematologic malignancies and neutropenia

**DOI:** 10.1038/s41598-020-73556-5

**Published:** 2020-10-05

**Authors:** Emese Tolnai, Gábor Fidler, Róbert Szász, László Rejtő, Kingsley Okechukwu Nwozor, Sándor Biró, Melinda Paholcsek

**Affiliations:** 1 Department of Human Genetics, Faculty of Medicine, University of Debrecen, Egyetem tér 1, Debrecen, 4032 Hungary; 2 Division of Haematology, Institute of Internal Medicine, Faculty of Medicine, University of Debrecen, Debrecen, Hungary; 3 Department of Hematology, Jósa András Teaching Hospital, Nyíregyháza, Hungary

**Keywords:** Biomarkers, Diagnostic markers, Predictive markers, Prognostic markers

## Abstract

Fungal infections represent a worrisome complication in hematologic cancer patients and in the absence of disease specific symptoms, it is important to establish new biological indicators, which can be used during mould-active prophylaxis. Recently, miRNAs have appeared as candidate diagnostic and prognostic markers of several diseases. A pilot clinical study was performed to evaluate the diagnostic utility of 14 microRNAs which can be related to invasive fungal infections. Based on our data miR-142-3p, miR-142-5p, miR-26b-5p and miR-21-5p showed significant overexpression (*p* < 0.005) due to invasive aspergillosis in hemato-oncology patients with profound neutropenia. A tetramiR assay was designed to monitor peripheral blood specimens. Optimal cut-off was estimated by using the median value (fold change 1.1) of the log10 transformed gene expressions. The biomarker panel was evaluated on two independent sample cohorts implementing different antimicrobial prophylactic strategies. The receiver operating characteristic analysis with area under the curve proved to be 0.97. Three miRNAs (miR-142-5p, miR-142-3p, miR-16-5p) showed significant expression alterations in episodes with sepsis. In summary, the tetramiR assay proved to be a promising diagnostic adjunct with sufficient accuracy and sensitivity to trace invasive aspergillosis in hemato-oncology patients.

## Introduction

In the developed countries life-threatening invasive fungal diseases (IFD) represent crude morbidity and mortality^1^. Members of the *Aspergillus* and *Candida* genera are predominant etiological agents^2,3^. In the past decades the incidence of aspergillosis has increased^4–7^. Currently, other relevant fungi are also increasingly identified, such as *Cryptococcus* spp., *Fusarium* spp. and, *Zygomycetes*^8^. Invasive aspergillosis (IA) can be frequently associated mainly with acute myeloid and lymphoid (AML, ALL) leukemia and occurs primarily in the lung^9^. The well-established risk factors include underlying malignancy, immunosuppression and profound neutropenia^10–12^.

Without timely initiated antifungal therapy the mortality of IA related hospitalization can top 70%, whereas in bone-marrow transplant patients, it has been reported to be even higher^13–15^. As a result of prolonged hospital stay, the burden of aspergillosis-related IFDs remains an immense public health problem, with an estimated treatment cost above $600 million/year in the US^13^.

In high-risk hematology patient groups, the first-line prophylaxis is preferred^16^. Timely initiation of treatment critically affects the outcome of the disease however diagnostic driven strategy remains a challenge^6^. Identification of IA relies on the combination of cultivation and non-conventional microbiological tests such as fungal antigen testing, as well as radiology and histopathology^4^.

MicroRNAs (miRNAs) are among the class of small, conserved, non-coding RNAs. By estimates, approximately 50% of the human transcriptome is influenced by miRNAs^17^. MiRNAs emerged as important endogenous regulators of virtually all basic biological processes^18^. In response to pathological processes miRNAs are expressed by all cell types and tissues into the circulation where their free forms are protected from RNAse mediated degradation^19^. Recent studies estimate the potential of free circulating miRNAs as prognostic disease biomarkers in diagnosis. During infection, lung hypoxia established by an ischemic microenvironment, vascular invasion, thrombosis, antiangiogenic factors such as gliotoxin, are considered to be a significant virulence factor of *A. fumigatus*, therefore hypoxia related miRNAs (miR26a, miR26b, miR21 and miR101) were also considered^20–22^. Further data revealed the participation of miR26a, miR26b in relation to apoptosis and autophagy^23^. The regulatory role of miR132 and miR155 was elucidated during *A. fumigatus* infection on human dendritic cells suggesting a strong involvement of these microRNAs in the anti-fungal response of the host^20^. MiR140, miR16 and miR494 were related to immune responses, anti-inflammatory processes accompanying infection, and cell apoptosis during *C. albicans* infection^23–26^. Furthermore, miR155, miR26, miR16 and miR21 were found to modulate immune response against Gram-positive bacteria in human, mouse and murine models^27–29^. However, due to the lack of consensus in methodology, as well as processing and normalization, the actual number of clinical studies which establish the contribution of certain miRNAs to IFDs is surprisingly low^20,21,30–35^.

To ameliorate success and accuracy of diagnosis, a definite proof-of-concept was set to capture invasive aspergillosis specific miRNA expression patterns in a hemato-oncology patient population. 14 miRNAs were chosen which are principally related to hypoxic microenvironmental stress conditions, inflammation, apoptosis and, autophagy^20–22,24–26,34,35^. All miRNAs are potentially obtainable from the peripheral blood circulation.

To our present knowledge, this is a pioneer clinical study which could lead to the implementation of miRNAs supporting the diagnosis of IA among patients with hematologic malignancies and profound neutropenia.

## Results

### Characteristics of patient cohort

**Figure 1:**
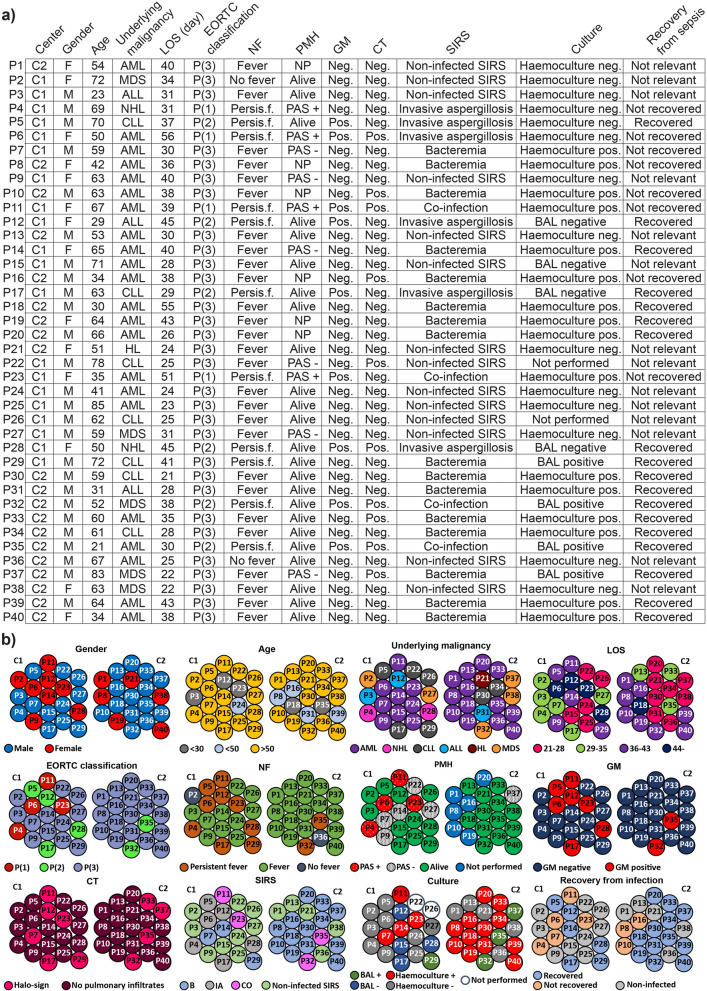
Shortlisting of parameters of our patient recruitment strategy with regard to baseline demographics and center specific characteristics. Demographic and clinical characteristics of patients with regard to their EORTC/MSG classification are described in (**a**) Center specific characteristics are shown in (**b**). C1 (center 1 Debrecen); University of Debrecen, Faculty of Medicine, Institute of Internal Medicine, Debrecen, Hungary. C2 (center 2 Nyíregyháza); Institute of András Jósa County and Teaching Hospital, Division of Hematology, Nyíregyháza, Hungary. (P1) proven IA, (P2) probable IA, (P3) possible IA-having no evidence of infection. AML Acute Myeloid Leukemia, ALL Acute Lymphoblastic Leukemia, CLL Chronic Lymphocytic Leukemia, MDS Myelodysplastic syndrome, HL Hodgkin Lymphoma, NHL Non-Hodgkin Lymphoma. EORTC European Organization for the Research and Treatment of Cancer/Mycosis Study Group, NF neutropenic fever, LOS Length of hospital stay, PMH Postmortem Histology, PAS Periodic acid–Schiff, CT Thoracic Computed Tomography, SIRS Systemic Inflammatory Response Syndrome.GM galactomannan serology, B bacteremia, IA invasive aspergillosis, CO co-infection, BAL bronchoalveolar lavage.

The vast majority of participants suffered from acute myeloid leukemia (C1 45%, C2 65%) and chronic lymphocytic leukemia (C1 25%, C2 10%) (Fig. 1a). 16 patients (40% of patients in both C1 and C2) died during the study period. Patients of these study groups were well balanced according to age (mean 60 ± 16.2, *p* = 0.229) and gender (mean 53 ± 15.8 *p* = 0.638). Overall, 95% of the onco-hematologic (OH) patients (except P2, P36) developed systemic inflammatory response syndrome (SIRS). 26 patients developed sepsis while 35% of patients had non-infected SIRS. Postmortem histology (PMH) was pursued in 56.25% of fatalities. In the case of four C1 patients (P4, P6, P11, P23), histological evaluation clearly proved the existence of IA. Descriptive Periodic Acid Schiff (PAS) and Hematoxylin and Eosin (H&E) stained hyphae supported the fungal invasion and the diagnosis of invasive aspergillosis. There was one case (P4) where pathology confirmed the presence of the fungus *Aspergillus* in the central nervous system. In C1 15.78% of patients suffered from both diabetes and hypertonia. Altogether, there were 11 patients (45% in C1, 10% in C2) with recurrent fever, refractory to antibiotic treatment and among these 4 patients (2 in C1, 2 in C2) were diagnosed with co-infections (CO: aspergillosis and bacteremia), 6 patients (all in C1) with invasive aspergillosis (IA) and 1 patient with bacteremia (B) (Fig. 1b). There were no significant differences in the inflammation status of the two study groups. Patient C-reactive protein levels in C1 (CRP: 107.57 ng/ul; inter quartile range (IQR) 55.89–159.25) and in C2 (CRP: 108.43 ng/ul; IQR 55.43–161.43). Patients were retrospectively categorized with P(1) proven IA, P(2) probable IA (25% cases) versus P(3) possible IA (having no evidence of infection). 100% of P(1) patients (4 cases) were diagnosed in C1 with 66.7% of P(2); 4 of 6 probable IA cases.

### First-line antifungal prophylaxis was associated with lower IA prevalence

In C2, primary antifungal prophylaxis was initiated in the case of OH patients. Relevant endpoints of this prophylactic strategy manifested with a sharp decrease in the number of IA cases (40% vs. 10%; *p* < 0.001) with no observation of proven IA (Fig. 1). On the contrary, in C2, significantly higher proportion of patients (13 patients, 65%) developed bacteremia in comparison to C1 (15%). The number of co-infections was balanced (10%) between the two centers.

### Identification of normalizer miRNAs

**Figure 2:**
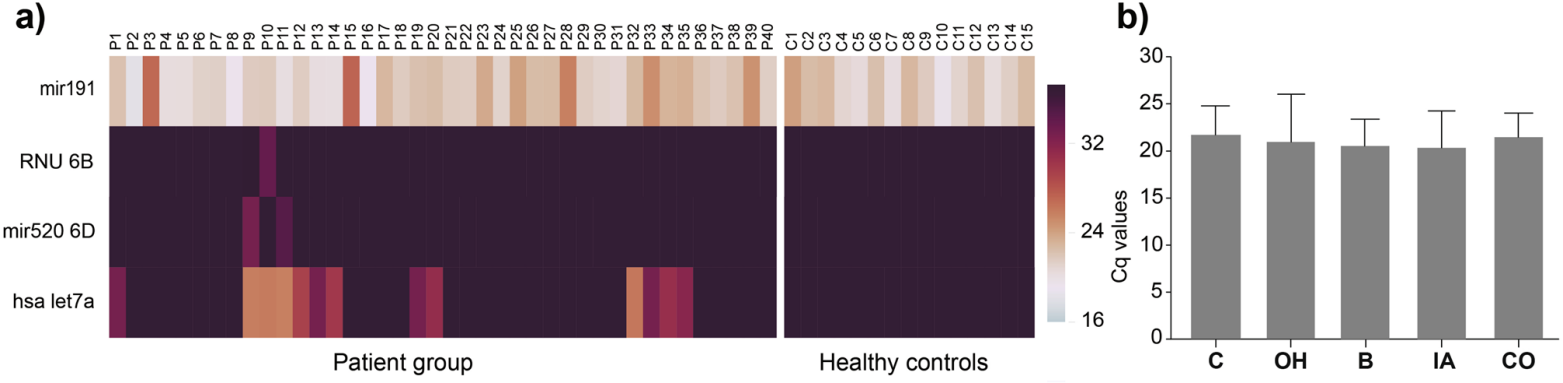
Testing of four potential miRNA normalizers. Hsa-miR191 was found to be optimal normalizer for estimating relative miRNA expression fold changes. (**a**) Heatmap represents arrayed Cq values for the normalizers on this cohort. P stands for onco-hematology patients of this study (Cq 21.34 ± 4.57), C stands for healthy controls (Cq 21.71 ± 3.07). (**b**) represents mean has-miR191 Cq values (± SD) in healthy controls (C) with no malignancies in comparison to OH patients (Cq 20.94 ± 5.1) developing B bacteremia (Cq 20.54 ± 2.84), IA invasive aspergillosis (Cq 20.34 ± 3.95) and CO co-infections (Cq 21.45 ± 2.56).

Initial miRNA testing was performed to identify an appropriate miRNA normalizer showing consistent gene expressions in the members of this study. MiR191 was found to be optimal across patients and controls (Fig. 2).

### Calculation of miRNA expression fold changes

**Figure 3:**
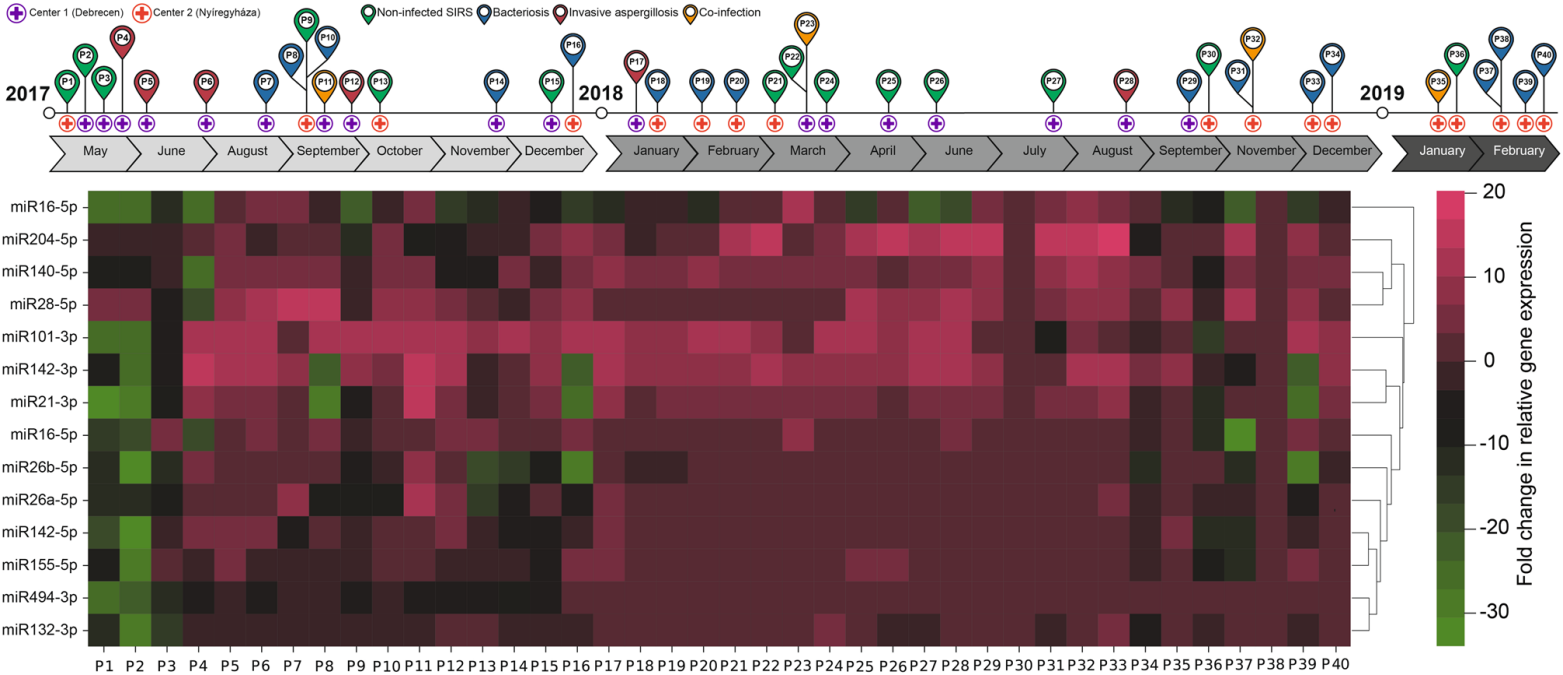
Relative expression levels of selected miRNAs in patients with hematologic malignancies. The expression levels of selected miRNAs detected by qRT-PCR were normalized to hsa-miR191 and presented as fold-changes (2^−ΔΔCt^). Black colors indicate no difference in gene expressions in comparison to the healthy controls, green scale represents the fold changes of under-, while pink the overexpressions.

Real-time quantitative PCR (qRT-PCR) was used to measure the relative gene expression levels of miRNAs in OH patients. Figure 3 displays the study timeline and the date of recruitments in the case of two Hungarian centers; C1 and C2 with a cluster heatmap demonstrating normalized relative gene expression levels of miRNA targets measured in the peripheral blood. All 14 miRNAs were expressed in the peripheral blood samples of patients and were generally overexpressed in comparison to healthy controls.

### Circulating miRNAs showed aberrant expressions in patients with sepsis

We managed to establish miRNAs showing significant expression alterations in infected SIRS patients. Based on our data three miRNAs were related to sepsis in OH patient population on the basis of significant changes in their relative gene expressions in the blood; miR142-3p (*p* < 0.01), miR142-5p (*p* < 0.05), miR16-5p (*p* < 0.01).

### Identification of aspergillosis specific miRNAs

**Figure 4:**
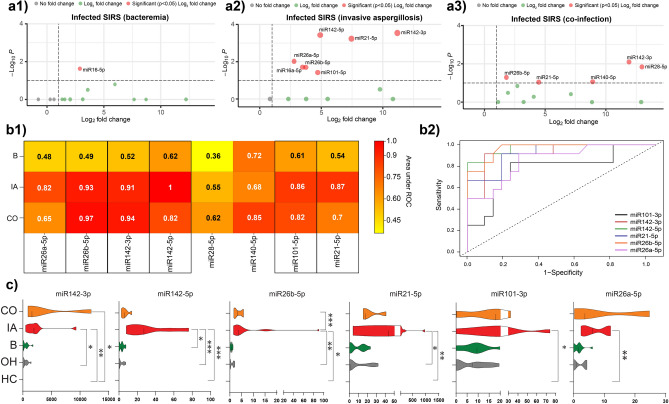
miRNAs showing statistically significant overexpressions indicating aspergillosis. Volcano plots were made to identify differentially expressed miRNAs due to aspergillosis using the LIMMA statistical model (**a**). Red spotted miRNAs represent statistically significant (*p* < 0.001) gene expressions indicating bacteremia (**a1**), aspergillosis (**a2**), bacterial and fungal co-infections (**a3**). Vertical lines represent miRNAs with a fold change of > 1.5. Red spotted microRNAs situated towards the right top quadrant represent values of increasing magnitude fold changes. CO stands for co-infection, IA invasive aspergillosis, B bacteremia, OH onco-hematology, HC healthy controls. (**a**) Volcano plots show log2 transformed fold changes in relation to − log10 transformed *p*-values. On the y-axis dashed lines indicate the *p*-value = 0.05, miRNAs with statistically significant expressions are shown with red spots. (**b**) ROC (Receiver Operating Characteristics) curves with AUC (Area Under the Curve) were made to check and visualize the performance of the 10 pre-selected miRNA assays. To measure the diagnostic accuracy of the miRNAs relative fold changes were converted to qualitative (proven IA, probable IA vs. possible IA) indexes. (**c**) Horizontal violin plots visualizing the distribution of the relative fold changes with probability density in different patient groups. *p ≤ 0.05; **p ≤ 0.005; and ***p ≤ 0.0005.

Nine miRNAs (miR142-3p, miR21-5p, miR142-5p, miR26a-5p, miR26b-5p, miR101-3p, miR16-5p, miR140-5p, miR28-5p) were overexpressed presenting statistically significant fold changes (*p* < 0.0001) in comparison to non-infected SIRS. In the case of miR16-5p significant overexpression was observed in both aspergillosis and bacteremia, thus it was omitted from further analysis (Fig. 4a1–a2). In the case of three miRNAs (miR26b-5p, miR21-5p, miR142-3p), bacteremia did not obscure the significant overexpressions indicating aspergillosis in diseased patients (Fig. 4a3). Only two microRNAs (miR28-5p, miR140-5p) showed significant overexpressions in cases with co-infections (Fig. 4a3). In Fig. 4b1 heatmap represents area under the curve (AUC) values estimated for 8 pre-selected miRNAs showing significant associations with infections in OH patients. Six microRNAs; miR26a-5p, miR26b-5p, miR142-3p, miR142-5p, miR101-3p, miR21-5p demonstrated good discriminatory property with high AUC values indicating aspergillosis. In Fig. 4b2 receiver operator curves (ROC) are also shown for miRNA discriminators, displaying AUC values greater than 82% in patients with aspergillosis. Reassuringly, the relative fold changes of these miRNAs proved to be specific for aspergillosis and bacterial co-infections did not interfere with the expression profiles (*p*-values 0.54–0.99) (data not shown). Violin plots with Kernel density estimations were also made to visualizing the distribution and probability density of relative fold changes of the miRNAs in different patient groups (Fig. 4c). In the case of miR26a-5p and miR101-3p we did not observe statistically significant (*p* ≥ 0.05) gene expression alterations between OH patients whether aspergillosis was diagnosed or not. With the considerable heterogeneity in the distribution of results mir142-5p provided significant overexpression in comparison to the non-infected patients (×80 ± 12; *p* < 0.0001), and its expression was significantly higher due to aspergillosis in comparison to bacteremia (×70 ± 14; *p* < 0.001). MiR26b-5p proved to be a promising aspergillosis specific biomarker (*p* < 0.0001).

### Diagnostic performance of the miRNA assays

**Figure 5:**
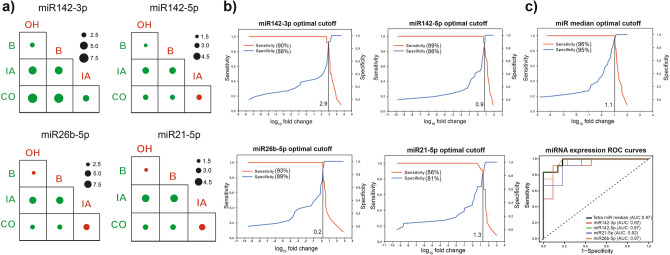
Log-2 transformed miRNA expression fold changes were used to exploit expression alterations between patient groups. (**a**) OH onco-hematology, IA invasive aspergillosis, B bacteremia, CO co-occurrence of bacteremia and aspergillosis. Extents of logarithmic (Δlog2fc) transformed fold differences are designated by dots. A dot colored red is more abundantly expressed by the condition designated in the columns while green colored dots show more abundant fold changes designated by the rows. (**b**) Optimal cut-point values were also estimated for mir142-5p (log10 fold change 0.9), miR26b-5p (log10 fold change 0.2), miR21-5p (log10 fold change 1.3), miR142-3p (log10 fold change 2.9) biomarker assays. (**c**) Estimating the optimal cut-point for tetramiR panel with ROC curve and with AUC to visualize performance of the method.

Differential expression matrices represent the values of alterations in relative miRNA fold changes (fc) when comparing different patient groups. MiR142-3p showed the highest association with aspergillosis (Δlog2fc 6.81) in OH patients compared to healthy controls (Fig. 5a). All miRNAs were assessed to be more abundant during aspergillosis and showed only a mild correlation with bacteremia. Noticeably, miR26b-5p (Δlog2fc: 4.28) and miR21-5p (Δlog2fc: 4.27) were expressed with a lesser extent during bacteremia. All miRNAs showed remarkable expression alterations due to aspergillosis in comparison to bacteremia. We observed moderate fold changes in the expression of several miRNA targets due to aspergillosis (mir142-5p Δlog2fc: 1.92, miR26b-5p Δlog2fc: 2.02; miR21-5p Δlog2fc: 1.67), in comparison to other infections, however mir142-3p was over-represented as per co-infections (Δlog2fc 6.37). Log10 transformed fold changes (log10 fc) for cases (proven IA, probable IA) and controls (possible IA) were dichotomized by mapping the sensitivity values in relation to 1—specificity in the case of mir142-5p, miR26b-5p, miR21-5p, miR142-3p to estimate optimal cut-off values for these biomarkers (Fig. 5b). MiR-21-5p (log_10_ transformed fold change: 1.3) showed the lowest, while miR-26b-5p (log_10_ transformed fold change: 2,9) showed the highest sensitivity (93%) and specificity (89%). To mitigate interpatient variability tetramiR diagnostic panel was established for a more reliable diagnosis of IA by the combined application of miR142-3p, miR142-5p, miR26b-5p, miR21-5p (Fig. 5c). This model relies on the median gene expression values (log10 fold change: 1.1) of these four miRNAs achieving higher sensitivity (96%) and specificity (95%).

### The diagnostic power of the tetramiR panel compared to the gold-standard Platelia *Aspergillus* GM-EIA

**Figure 6:**
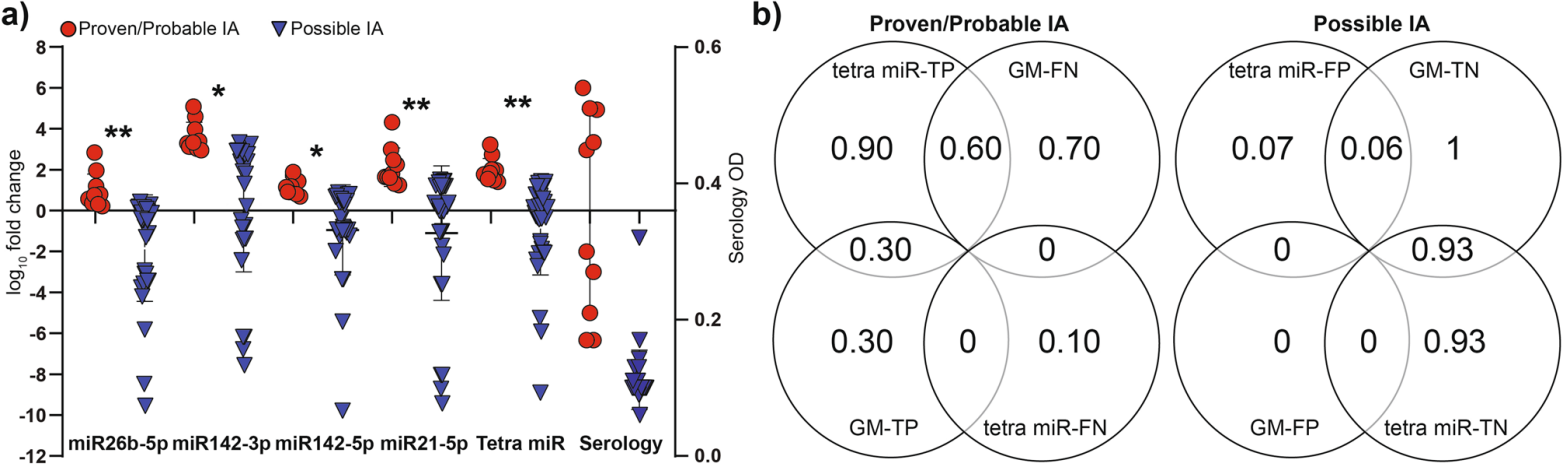
Diagnostic utility of the tetramiR panel when compared to the *Aspergillus* GM-EIA method. (**a**) Values of the test results categorized to proven IA, probable IA, possible IA-having no evidence of infection and their distributions shown with significances. *p ≤ 0.05; **p ≤ 0.005; and ***p ≤ 0.0005. In the case of tetramiR panel the median of the log10 transformed gene expression data was used. (**b**) Venn-diagrams with section-analysis to compare the outcomes of Platelia *Aspergillus* GM-EIA and tetramiR qRT-PCR panel diagnosing invasive aspergillosis. TP true-positive, FN false-negative, TN true-negative, FP false-positive. Cases (proven and probable IA) with positive GM-EIA and tetramiR panel results were regarded as true positive (TP) while those with negative outcomes were considered to be false negative (FN). Controls (unclassified patients with no EORTC/MSG evidence of IA) with negative GM-EIA and tetramiR panel results were coded to true negative (TN) and those with positive outcomes false positive (FP).

To compare the performance of the tetramiR panel to the gold-standard Platelia *Aspergillus* GM-EIA test sensitivity, specificity values were estimated for both classifiers on this patient population. Distribution of the test results of cases and controls are also shown with significances (Fig. 6a). Sample concordance was also assessed between the two classifiers; Platelia *Aspergillus* GM-EIA and tetramiR panel showed a fair agreement with an observed ratio of 77.5% (agreement; 31 of 40) and a kappa statistic of 0.41 (100% confidence interval from 0.1672 to 0.6713). We did not observe significant difference in the distribution of test results between cases and controls. Out of the 10 patients diagnosed with IA 30% proved to be true positive (TP) with both of the methods. GM-EIA failed to detect IA in 7 of 10 (TP 30%) and tetramiR panel 1 of 10 (TP 90%) cases (Fig. 6b). None of them provided false negative (FN) results. 60% of IA cases were detected only by tetramiR assay. The specificity of GM-EIA was 100% providing no single FP results among controls whereas the true positivity rate was 93% in the case of the tetramiR assay.

## Discussion

With the significant developments achieved in the field of oncology, nosocomial etiologies are continuously associated with high mortality and increased susceptibility to IFDs^12^. The prophylactic use of available azoles during anticancer therapy has led to a reduction of *Candida* blood stream infections however the growing antifungal resistance especially in the case of *Aspergillus fumigatus* and *A. terreus* has emerged as a global health issue^12,36–41^. In Europe the genus *Aspergillus* accounts for 12–28% of all fungal isolates^41–44^. *Aspergillus*-related pulmonary diseases can encompass various clinical syndromes moreover immunosuppressed patients may develop invasive pulmonary aspergillosis (IPA)^45,46^.

Despite the immense number of published guidelines, no obvious recommendations are available addressing diagnosis and managing infections in these patient groups^47–52^.

This pilot study was performed to capture the expression of specific free circulating miRNAs in blood to support the diagnosis of invasive aspergillosis. Among patients with hematologic malignancies and profound neutropenia IA was diagnosed in 14% of episodes and 55% of these cases were diagnosed post-mortem only. Owing to the fact, that the vast majority of patients suffered by AML and CLL (in total: 72.5%) this study population can be considered homogenous in terms of underlying malignancies.

In the case of severely immunocompromised patients the escalation in the lengths of hospital stay (LOS) appreciably raises the odds of nosocomial co-infections. Patients with fungal septicaemia show significantly higher mean hospital costs thus duration of hospitalization was positively correlated with disease occurrence^53^. According to our results none of the study participants with LOS < 29 developed IA. Whereas the highest disease prevalence (80%) was observed in patients with LOS ≥ 44, while IA was diagnosed in 27% of patients with LOS ≥ 29, LOS < 36.

Sepsis is a systemic inflammatory response syndrome (SIRS) driven by infection and is a leading cause of mortality in patients after intensive cytotoxic therapy significantly decreasing the survival rate with more than 50%^12,54,55^. The prevention of infection has become a major goal however there is no widely accepted standard for antimicrobial prophylaxis^17,27,56^^.^ The risk appreciably rises in severe neutropenic episodes having persistent fever with more than 7 days. Based on overlapping data of previous studies the induction of miR132, miR155, miR26, miR16 and miR21 was found to modulate immune response to different bacterial stimuli^28,29,57–62^. These estimations did not manifest in our data with one exception. The contribution of hsa-miR16 to bacterial infections was strengthened showing significant expression alterations.

A difference was shown in the distribution (C1 47.36%, C2 only 10.52%) of episodes in whom fever persisted despite antibiotics. An appreciable rise was observed in the number of aspergillosis patients who did not receive antifungal prophylaxis. In C1 remarkably higher proportion of SIRS episodes (77%) developed sepsis, out of which the vast majority (85.71%) was diagnosed with IA.

Antimicrobial prophylaxis has already been estimated as an important prognostic factor of bacteremia in several studies^12,63–70^. Due to a more intense antifungal prophylactic strategy in C2 there were only two episodes (P35, P32) with recurrent fever developing non-infected SIRS. A paradox was found when comparing the number of infection related SIRS between C1 and C2 and the onset of diagnosed bacterial infection and invasive aspergillosis. The prevalence of aspergillosis proved to be remarkably lower in the case of C2, in comparison to C1 (10% vs. 60%; *p* < 0.001), which can be explained by the more intensive prophylaxis management followed by C2. On the contrary, in C2, significantly higher proportion of patients (13 patients, 65%) developed bacteremia in comparison to C1 (15%).

Numerous studies have been conducted in recent years to establish expression levels of certain miRNAs in different disease conditions^4,53^. With the fact that miRNAs are expressed by all cell types and tissues they are often present at very low concentrations in peripheral blood thus their quantification requires highly sensitive and specific methods. Diverse technical biases such as initial sample volume, collection modality and, storage condition can be introduced during the experimental steps which profoundly obscure the true biological response^71–75^. Additionally, miRNA family members exhibit a high degree of homology having rather low absolute copy numbers in body fluids. Peripheral blood samples are enriched with both free circulating, membrane enclosed and intracellular miRNAs as well.

The choice of a reference gene remains problematic, having serious impact on the available transcript levels and consequently, on the adequate interpretation of data. We therefore applied and tested different assays to find a reliable normalizer to analyze circulating miRNA expression profiles. Hsa-miR191 proved to be an appropriate reference miRNA to mitigate inter-patient variabilities. Exogenous influences and the genetic heterogeneity which is concomitant with haematological malignancies can obscure the miRNA regulome. To mitigate these accidental effects, we recommend to draw conclusions from the expression alterations of multiple biomarkers. In the case of four miRNAs (miR142-3p, miR142-5p, miR26b-5p and miR21-5p), a significant association to IA was confirmed. We therefore decided to label this set of miRNAs as a novel biomarker panel (tetramiR panel) supporting diagnosis of invasive aspergillosis. We found, that the expression of these key indicators was not confounded by age, gender, prophylactic drug choice, underlying malignancy, other diseases (diabetes, hypertonia) and sepsis. Considering the fact that our sampling data reflect only single time points conclusions cannot be drawn about how the mirRNA expression profiles correlate with disease progression.

The diagnostic power and the accuracy were estimated for the tetramiR panel and compared to the Platelia *Aspergillus* GM-EIA method. In 66.66% of the patients with pulmonary infiltrates the Platelia *Aspergillus* GM-EIA showed a favorable diagnostic accuracy which is in line with observations of other studies^76^. In the case of the galactomannan-enzyme immunoassay (GM-EIA) studies steadily report about a various test performance (positive predictive values: 25–62% and negative predictive values: 92–98% using 0.5 as the cut-off)^77,78^. By monitoring the hemato-oncology episodes with profound neutropenia the results of the GM-EIA exerted poor sensitivity in comparison to the tetramiR panel (GM-EIA Se: 30% vs. tetramiR panel: 90%).

Diagnosis of invasive aspergillosis still constitutes a major issue in the severely immunocompromised patient groups, especially in those undergoing intensive cytotoxic therapy. Based on our data, the tetramiR panel which relies on the expression of four concomitant miRNAs (miR142-3p, miR142-5p, miR26b-5p and miR21-5p) can serve as a good adjunct in the diagnosis of aspergillosis. Although the optimal diagnostic cut-off point of this method might be biased by the limited size of the patient population, we underpin its suitability and utility in real diagnostic practice.

## Materials and methods

### Patient population

This retrospective case–control study was performed from May 2017 to February 2019, involving two hematology centers in Hungary: University of Debrecen, Faculty of Medicine, Institute of Internal Medicine, Debrecen, Hungary (C1) and Institute of András Jósa County and Teaching Hospital, Division of Hematology, Nyíregyháza, Hungary (C2). Patient population comprised 40 adult admissions; 26 males with the median age of 52 (range 21–83) and 14 females with the median age of 54 (range; 24–83) having different hematological malignancies (mainly acute leukemia—42%) receiving stem cell transplantation and intensive chemotherapy (neutrophil count < 0.5 × 109 cells/L). Patients developing neutropenic fever (NF); temperature > 38 °C of fever recorded twice or > 38.5 °C recorded once were recruited with two exceptions (P2, P36). Children < 17 years were excluded from the study. There were 15 healthy controls included with no previous history of onco-hematology (OH) diseases or SIRS with the median age of 42.5 (range 24–75).

### Diagnosis of sepsis

Patients were considered to develop SIRS presenting minimum 2 of the following clinical findings; (1) body temperature less than 36 °C (96.8 °F) or greater than 38 °C (100.4 °F), (2) heart rate greater than 90 beats per minute, (3) tachypnea greater than 20 breaths per minute; or, an arterial partial pressure of carbon dioxide less than 4.3 kPa (32 mmHg), (4) white blood cell count less than 4000 cells/mm^3^ (4 × 10^9 ^cells/L) or greater than 12,000 cells/mm^3^ (12 × 10^9 ^cells/L). Bacterial and/or fungal sepsis was defined using clinical and laboratory parameters such as culture-based pathogen detection with the isolation of viable microorganisms from blood cultures^79–83^.

### First-line choices for antimicrobial prophylaxis

C1 (Debrecen) and C2 (Nyíregyháza) followed two different antimicrobial prophylactic strategies. Both of the centers initiated antiviral and antibacterial prophylaxis in hematology and oncology patients in parallel with the administration of chemotherapeutics^84–86^. In C2, antifungal therapy was uniformly initiated immediately at the onset of neutropenia while in C1 only 50% of the cases received prophylactic antifungals, and in the rest of the cases, therapy was administered only in the case of patients with fever refractory to broad-spectrum antibiotics (≥ 6 days of fever).

### Stratification of episodes

Patients were retrospectively stratified using standard criteria according to revised European Organization for the Research and Treatment of Cancer/Mycosis Study Group (EORTC/MSG) to define a case-group of “P(1) proven” or “P(2) probable” IA (10 patients, 25%) and a group of patients comprising P(3) possible IA (30 patients 75% with no EORTC/MSG with no evidence of infection)^87,88^.

### Sampling

Patient samples came from 40 prospectively enrolled hemato-oncology patients from two centers; C1 and C2 of Hungary. For galactomannan testing and PCR analyses peripheral blood samples were collected from every patient at the onset (day 1) of fevered neutropenia. In the case of P2 and P36 samples were collected from non-fevered neutropenic patients suspected to develop fungal infections. In 95% of the cases (except P22, P26), hemocultures (75%) or BAL samples (20%) were also obtained in parallel the collection of blood samples.

### Cultures

There were 30 cases where hemocultures were obtained. Adult patients having bacteremia typically have low quantities of bacteria in the blood, therefore multiple blood culture examinations were performed (C1: 2.07 ± 1.98, C2: 3.33 ± 3.2) to detect blood stream bacterial infections. Classical mycology of bronchoalveolar lavage (BAL) was performed in the case of 8 patients in whom there was a suspicion of bacteremia or aspergillosis (infected SIRS).

### Biomarker analyses

Liquid biopsy samples were parallelly screened for the presence of galactomannan (GM) antigens and for microRNA. Intact EDTA serum tubes were drawn from patients routinely for GM analysis in parallel with intact EDTA whole blood tubes for microRNA analyses and sent to the Department of Medical Microbiology, Debrecen, Hungary.

### Platelia *Aspergillus* GM-EIA

The GM-EIA assays required 300 μl of serum specimens. The Platelia *Aspergillus* GM-EIA (Bio-Rad Laboratories, Hungary) was used for GM screening. GM-EIA cut-off values were determined using the OD_450/620_ values. While evaluating single specimens any value above the OD (optical density) _450/620_ ≧ 0.5 cut-off value was considered positive as requested for in vitro testing.

#### miRNA-based biomarker assays

Whole blood tubes were forwarded to the Department of Human Genetics, Debrecen, Hungary. MicroRNA analyses were carried out in a class II laminar-flow cabinet to avoid environmental contamination. Total RNA was extracted from the peripheral blood using the MagMax mirVana according to manufacturer’s instructions (Thermo Fisher Scientific, Maryland) by recovering on average 46.3 ± 42.8 SD ng RNA/µl from 50 µl peripheral blood specimens. 3 ng of RNA was constantly used for the miRNA specific reverse transcription using TaqMan Advanced miRNA cDNA Synthesis Kit (Thermo Fisher Scientific). To identify a stabile miRNAs endogenous control in whole blood samples of healthy patients and in study participants four candidate miRNAs (RNU6B, miR520-6d, hsa-let7a, miR191) were selected on the basis of literature data^73,89^. TaqMan quantitative real-time PCR (miRNA assay) was used to detect miRNA expression profiles in 3 independent technical repeats including negative controls (no-template from the RT reaction), using a LightCycler 96 Real-Time PCR System (Roche Diagnostics, Risch-Rotkreuz Switzerland). Normalized miRNA expression levels of 14 miRNAs (miR26a-5p, miR26b-5p, miR21-5p, miR142-3p, miR142-5p, miR16-1-3p, miR16-5p, miR155-5p, miR140-5p, miR101-3p, miR494-3p, miR28-5p, miR132-3p and miR204-5p) were measured with quantitative real-time PCR (qRT-PCR) assays. Relative mRNA expressions were calculated by using hsa-miR191 for normalization.

### Statistical analyses

Expression levels of the selected miRNAs detected by qRT-PCR were normalized to miR-191 and presented as the fold change (2^−ΔΔCt^) above the controls (non-HM): ΔCt = assay Cq—normalizer Cq. Results for non-normally distributed continuous variables are summarized as medians (interquartile ranges) and were compared by Mann–Whitney U test. Statistical comparison among multiple groups were performed with Kruskal–Wallis test, and intergroup differences were tested with Dunn test. Significance was accepted at *p*-value < 0.05. Levels of significance were assigned as: *p ≤ 0.05; **p ≤ 0.005; and ***p ≤ 0.0005. All statistical analyses were performed using GraphPad Prism statistical software. To measure the diagnostic accuracy for the GM-EIA and the tetramiR panel, results (GM-OD and normalized Ct) were converted to qualitative (positive, negative) indexes. In the case of the qRT-PCR based tetramiR panel every single run was evaluated independently prior to calculating the median of the Ct values to take diagnosis. Area under the ROC curve (AUC) was used as an accuracy index for evaluating the diagnostic performance of the selected miRNAs. After calculating the series of sensitivity (Se) and specificity (Sp) reports at every single decision threshold, the receiver operating characteristic (ROC) curve was edited by plotting the true positive values (sensitivity values on y axis) versus the false positive rates (1—specificity values on x-axis). Area under the ROC curve (AUC) was estimated with 95% confidence intervals (95% CIs) and with standard errors (± SD)^90^.

### Postmortem histology

The specificity of *Aspergillus* infection morphology via Periodic acid–Schiff (PAS) staining was addressed due to the open lung biopsy, performed via postmortem thoracotomy^91^. Histological samples were taken from the major organs according to a standard protocol. Lung sampling was performed from three independent parts of the potentially infiltrated lung parenchyma.

### Ethical statement

The study protocol was approved by the ethics committee of the University Hospitals of Debrecen, Hungary (MK-JA/50/0096-01/2017) and carried out in accordance with the approved guidelines. In the case of postmortem histology informed consent was obtained from the donor’s next of kin. Written consent was obtained from all participants. Measuring of miRNA expression levels were performed in parallel with *Aspergillus* GM-EIA.
